# Association Claims in the Sequencing Era

**DOI:** 10.3390/genes5010196

**Published:** 2014-03-11

**Authors:** Sara L. Pulit, Maarten Leusink, Androniki Menelaou, Paul I. W. de Bakker

**Affiliations:** 1 Department of Medical Genetics, Institute for Molecular Medicine, University Medical Center Utrecht, Universiteitsweg 100, 3584 CG, Utrecht, The Netherlands; E-Mails: s.l.pulit@umcutrecht.nl (S.L.P.); m.leusink@uu.nl (M.L.); a.menelaou@umcutrecht.nl (A.M.); 2 Division of Pharmacoepidemiology & Clinical Pharmacology, Utrecht Institute for Pharmaceutical Sciences, Utrecht University, Universiteitsweg 99, 3584 CG, Utrecht, The Netherlands; 3 Julius Center for Health Sciences and Primary Care, University Medical Center Utrecht, Universiteitsweg 100, 3584 CG, Utrecht, The Netherlands

**Keywords:** association, GWAS, next-generation sequencing, significance, bias, complex traits

## Abstract

Since the completion of the Human Genome Project, the field of human genetics has been in great flux, largely due to technological advances in studying DNA sequence variation. Although community-wide adoption of statistical standards was key to the success of genome-wide association studies, similar standards have not yet been globally applied to the processing and interpretation of sequencing data. It has proven particularly challenging to pinpoint unequivocally disease variants in sequencing studies of polygenic traits. Here, we comment on a number of factors that may contribute to irreproducible claims of association in scientific literature and discuss possible steps that we can take towards cultural change.

## 1. The Evolution of Genetic Association Studies

The Human Genome Project [1] remains the largest scientific endeavor in the biological sciences, spanning thirteen years, requiring hundreds of researchers around the globe, and costing $3 billion. Called the “most important, most wondrous map ever produced by human kind” [2] by U.S. President Clinton upon its completion, the map of the genome catapulted the investigation of human disease into a new era. The study of complex traits and disease had previously been limited to genetic linkage studies, typically laborious efforts limited to constructing linkage maps in families and powered for discovering highly penetrant variants. Now, geneticists could identify single-base changes in the genome (single nucleotide polymorphisms, or SNPs) and, by measuring the frequencies of these changes in large groups of cases and controls, test SNPs for association with susceptibility to any number of diseases.

Upon the completion of the Human Genome Project, the field of genetics was faced with determining how best to hunt for such associations. The first widely used method was candidate gene studies, in which genes with suspected biological or functional relevance to the disease in question were selected for testing. These studies were applied to a variety of traits [3], but in the absence of community-wide standards for analysis, they were plagued with problems. Reviews of candidate gene studies found that small sample sizes, population stratification issues, weak effects, and a lack of statistical evidence for the claimed associations were common [3,4,5,6]. All of these problems contributed to the irreproducibility of initial findings; one review found that of 166 associations with more than two follow-up studies, only six (3.6%) replicated [3].

Despite limited success in candidate gene studies, the number of replicated associations indicated that common variation (frequency >5%) indeed plays a role in genetic susceptibility to common disease (involving tens of genes and environmental factors) [7]. In 2005, the approach in genetics began shifting towards genome-wide association studies (GWAS) [8], which require no prior hypothesis about which genes are likely to influence disease. Instead, geneticists could test millions of common variants across the genome for association with the trait of interest. Systematic cataloguing of these variants [9] allowed for the design of genome-wide SNP arrays, ultimately allowing for low-cost capture of tens of thousands of variants in large samples.

**Figure 1:**
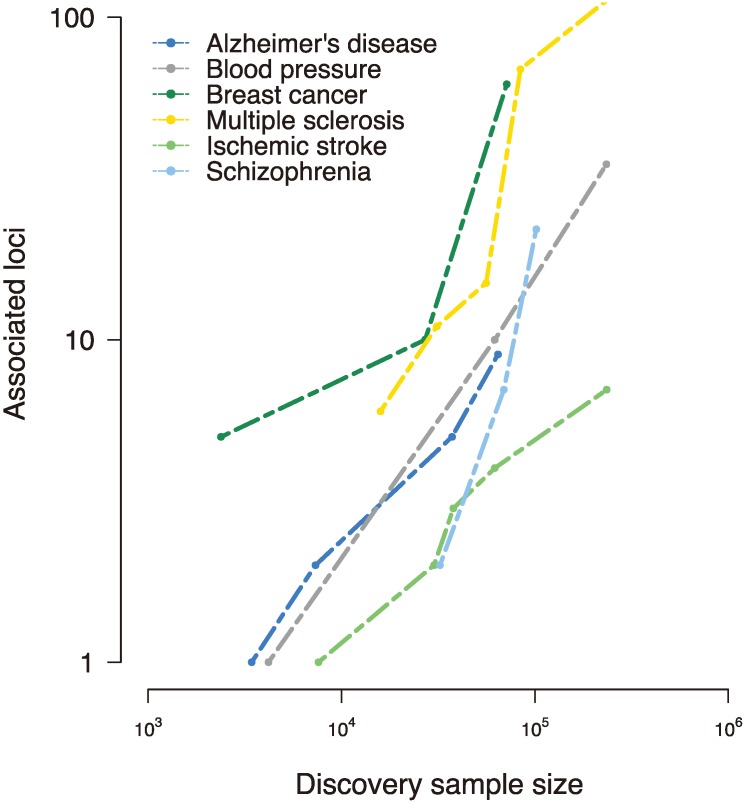
Disease-susceptibility loci discovered to date in various complex traits, as reported in the National Human Genome Research Institute (NHGRI) genome-wide association studies (GWAS) catalog [17]. Early genome-wide association studies interrogated small samples and uncovered few, if any, loci associated with the trait of interest. However, collaborative efforts to assemble large-scale samples improved power and implicated tens and even hundreds of susceptibility loci, revealing a (roughly) linear relationship between sample size and associated loci [18].

The beginnings of GWAS were slow; initial studies produced few if any associated loci, and it quickly became clear that larger samples would be necessary for sufficient power to detect susceptibility variants [10]. With the formation of international consortia, collection of large samples, and assembly of imputation panels that allowed for testing of variants not present on SNP arrays came an explosion in discovered loci (Figure 1). Along with the rapid increase in the number of GWAS being performed came a large-scale effort to standardize the method. The community adopted the genome-wide significance *p*-value threshold of 5 × 10^−8^, a *p*-value that reflects a Bonferroni correction for the approximately one million independent tests performed in a GWAS [11,12]. Methods for handling population stratification were developed [13], as were approaches for finding and removing poorly captured genotypes [14]. Replication of discovered loci also became a criterion for declaring a SNP to be associated with a disease [15,16]. With best practices in place, GWAS has become an efficient and robust method for discovering the contribution of common variation to susceptibility in common disease. The total number of SNPs associated with complex traits (at genome-wide significance) was only seven by the end of 2006, but by 2008, an additional 637 associations had been discovered [17]. Today, more than 6,000 disease-SNP associations have been reported.

Because of established best practices and statistical thresholds, most GWAS findings are robust and reproducible. The method is, of course, not immune to error; for example, one GWAS looking for variants associated with longevity failed to correct for technical bias introduced by the use of two different SNP arrays, inducing spurious association [19]. The mistake went unnoticed until the results were published and several geneticists caught the error. The false report received attention from the genetics community and the media alike [20] and was later retracted [21].

More recently, scientists and the media have cast a critical eye towards the aspects of scientific culture that also give rise to false-positive findings in published work. The *Economist* recently published an article suggesting that science is not as self-correcting as many assume [22,23], and journals such as *Nature* and *Science* are publishing columns on scientific misconduct, peer-review, and other issues that contribute to the reporting of inaccurate results [24,25,26,27,28]. The last four years have also seen several published studies on the prevalence of false discoveries and misconduct, indicating that as many as “1% of published papers are fraudulent” (about 20,000 papers each year) [25]. Given the increased attention from the public and scientists alike, and because genetics is in a transition phase as it moves from performing GWAS data to studying next-generation sequencing data, now is an ideal time to address some of these shortcomings.

## 2. Sources of Error and Bias in Genetic Research

A number of factors contribute to false-positive findings in published human genetic research. Technical artifact, such as mishandling of population stratification, poorly genotyped SNPs, and batch effects introduced by different SNP arrays or genotyping runs can cause spurious results, though a number of methods have been designed for detecting them [13,29,30,31,32]. Study design is also crucial in avoiding false positives. SNPs implicated in disease susceptibility typically have modest effect sizes (odds ratios ranging from 1.1–1.5) [18]; insufficient sample size to detect such effects can substantially reduce a study’s power and increase the likelihood of discovering an artifact rather than a true association.

Although technical error can lead to false positives, a number of other forces in research culture also contribute to the number of published erroneous findings. In the span of just seven years, GWAS moved from small-scale efforts to studies of thousands of samples, leading to a heavy reliance on statistical methods to study large datasets. Yet, researchers’ understanding of statistics has not kept pace with data generation, making them more likely to apply inappropriate statistical tests or perform tests they do not fully understand. The same holds true for the many programs written to analyze genome-wide datasets. Not all researchers using this software will fully understand the underlying methods, increasing the chance of false positives going unnoticed.

The peer-review process is also partially to blame for the introduction of false findings into scientific literature. Though researchers would seem ideal candidates for catching the mistakes of their peers, studies suggest that they often fail to catch errors (even when instructed that there are errors to find) [22]. Further, peer-reviewers are not provided all data underlying a paper and therefore cannot reproduce analyses to verify findings.

Studies also indicate that misconduct (which includes plagiarism, fraud, and duplicate publications) has been on the rise in recent years [33] and accounts for the majority of retracted papers in the life sciences [34]. A number of aspects of research culture likely contribute to such misconduct. Scientific research has become a field in which the number of articles a researcher produces is a primary measure of success. Career opportunities in the sciences also often hinge on the number of publications a scientist has produced. Conflating financial interests (whether in the form of employment or grants) and pressure to publish is a factor that potentially gives rise to false associations reported in scientific literature [35]. Anyone pressed to produce such a high volume of results, preferably at great speed, is more likely to miss mistakes in her own work.

Publication and funding bias are also problematic [36]. Although replicating a result is the backbone of establishing the veracity of a scientific claim, replication studies are less likely to be funded than discovery-focused experiments, and journals are less likely to publish them. Journals also typically do not publish negative studies, preferring to focus instead on novel results. The establishment of the impact factor system has further intensified journals’ bias towards novelty. While selecting manuscripts for publication, journal editors consider not only the content of the manuscript, but also the potential that the paper will improve the journal’s impact factor. The impact factor ranking system also heightens publishing competition for researchers. Researchers seek publication in only a very small set of highly selective journals in pursuit of many citations, widespread attention from the scientific community and popular media, and potential career advancement. In molecular biology and genetics, just six journals accounted for 85 of the 100 most-cited articles between 1998 and 2008 [37].

**Figure 2:**
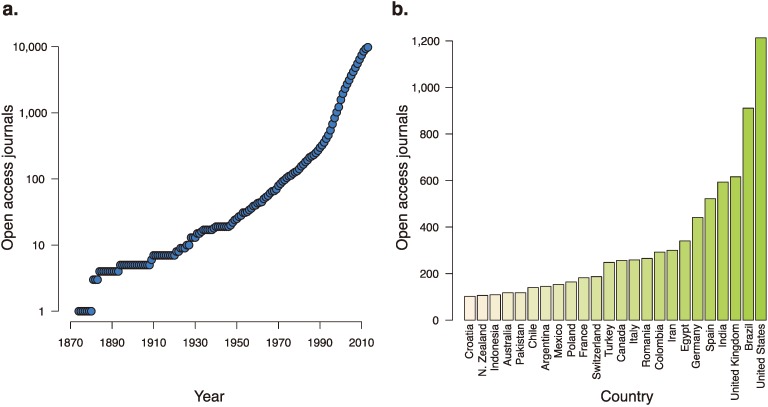
The growth of open access journals over time and around the world. (**a**) The number of open-access journals, as tracked by the Directory of Open Access Journals, over the last 140 years. (**b**) Countries with over 100 open-access journals accepting manuscripts.

Increasing the prevalence of false positives are those journals that seek to turn a profit by preying off a research culture that keeps publishing at its center. The number of journals accepting manuscripts is large and rapidly growing. The Directory of Open Access Journals, which tracks open access journals and their credibility, has tracked the swift global expansion of the number of open access journals, including an addition of over 1000 journals in 2012 alone [38] (Figure 2). A “sting” conducted on open-access journals revealed that many of them are uninterested in scientific veracity; a paper concocted wholesale and containing glaring errors was accepted by more than 52% of the targeted journals [39]. The “sting” in part implicated a flawed peer-review system, as 40% of the submissions were reviewed and then accepted. The other 60% of submissions, however, were accepted without any indication of peer review, suggesting that many of the journals the “sting” targeted are focused more on profit rather than on scientific rigor, encouraging a culture that values a published finding over a robust result.

## 3. Next-Generation Sequencing: New Technology, New Challenges

The advent of next-generation sequencing (NGS) technology has ushered in a new wave of studies in human genetic research. Given that many complex traits involve tens and sometimes hundreds of loci [40,41], lower-frequency variants may also contribute to the architecture of human disease. However, these variants are weakly tagged by common SNPs and have therefore gone untested by GWAS efforts. Researchers using NGS data can test (nearly) the entire set of variants in a single genome, helping to complete the picture of the role of genetic variation in common disease. Yet, this new technology brings with it many challenges, giving rise to additional forces that may lead to false positives in scientific literature.

A number of technical errors can give rise to a false-positive association in a NGS disease study. Determining genotypes from sequencing reads is more challenging than determining genotypes from SNP array data [42,43]. Rather than measuring probe intensities, as is done with SNP arrays to determine genotypes, extracting genotypes from sequencing data involves multiple steps, including mapping sequencing reads to a reference, detecting bases that do not match the reference, and determining the genotypes of each individual at each base; errors can occur at any of these steps and are particularly likely in regions that are difficult to capture, such as those rich in GC content or that are highly repetitive. Further, determining inclusion and exclusion criteria for variants based on a host of sequencing metrics can be difficult, sometimes requiring manual review of each of the metrics to determine appropriate filters [44]. Even the most conservative variant calling and quality control (QC) cannot guarantee that “variants” that are actually artifacts will be removed from the dataset.

Study design flaws can also prompt false-positive results. Although methods for detecting population stratification have been developed and widely used in GWAS, our understanding of population stratification in rare variants is limited and may confound association results [45]. To reduce costs, an investigator may choose to sequence only cases and use external (previously sequenced) controls; this approach may introduce stratification because the controls may be sequenced using a different platform [46] or genotype-called using outdated software. Alternatively, an investigator may want to sequence only cases and then genotype the discovered variants in controls or have cases substantially outnumber the controls, but these approaches also inflate type I error [47].

So-called loss-of-function mutations are of particular interest in NGS studies since they truncate proteins and are thus good candidates for likely pathogenic mutations. However, determining the deleteriousness of a loss-of-function mutation can be challenging, and this class of variants is enriched for artifacts [48]. It may be tempting to relax statistical thresholds for loss-of-function mutations or produce functional results for them before assembling appropriate statistical evidence from the genetic data, but doing so can lead to error.

**Figure 3:**
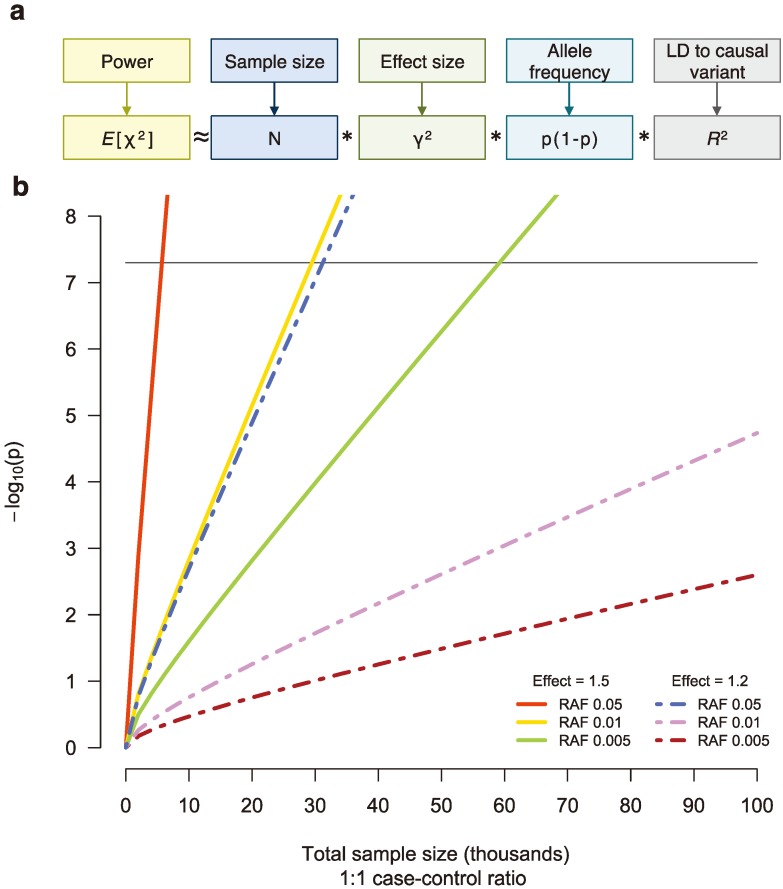
(**a**) The power to detect a genetic association is a function of sample size (*N*), effect size (γ), the frequency of the associated allele (p), and linkage disequilibrium (LD) between the tested and causal variants (*R*^2^), assuming an additive model [50]. (**b**) For sequencing studies, many thousands of samples will be necessary to detect single, low-frequency variants associated with disease risk at genome-wide significance (black line). RAF, risk allele frequency.

Though there are many challenges in performing an NGS study, the single largest problem plaguing sequencing disease studies to date is low statistical power to detect an association. The hypothesis that rare variants influencing susceptibility to common disease would have larger effect sizes than those discovered by GWAS has gone largely unsupported, and analyses indicate that NGS studies will require tens and even hundreds of thousands of samples to be well powered [46,49] (Figure 3). Consequently, assembling adequate sample sizes for NGS studies will take time, but researchers remain under pressure to publish. As a result, they will likely be anxious to push forward results that are not fully understood, lack statistical evidence, and may not even be real.

Finally, publishing bias will prove even more problematic in the sequencing age. The optimal choices for designing and performing a sequencing study—from phenotype ascertainment to selecting algorithms for calling and filtering variants and deciding which association tests to apply—remain difficult to discern. Sequencing studies that lack “positive” findings are highly informative but publishing bias will prevent such information from being widely disseminated. It will be more difficult to establish a standardized methodology for NGS studies, as was done for GWAS, because it is likely that “failed” experiments will not be given the same attention as studies reporting new and exciting results.

## 4. Lacking Evidence: Examples from NGS Studies

To publish disease-associated loci discovered through genome-wide association studies, it has become standard practice to meet basic criteria for discovery: appropriate sample and genotype cleaning, a SNP at genome-wide significance with a reasonable effect size and frequency, and replication in independent samples. Such standards do not yet exist for sequencing studies. Without criteria for claiming an association combined with publication bias and the pervasive pressure to “publish or perish,” some NGS studies in complex traits have been published despite a paucity of statistical evidence.

A targeted sequencing project in anorexia nervosa (AN) patients claims an association between AN and the epoxide hydrolase 2 (*EPHX2)* gene, though the burden test *p*-value of the gene failed to meet exome-wide significance (discovery, *p* = 4 × 10^−4^; replication, *p* = 6.2 × 10^−3^) [51]. Because *EPHX2* has been linked to lipid traits and hypercholesterolemia is common in AN patients, the authors performed a variety of interaction tests between variants in *EPHX2* and cholesterol and body mass index. They suggest that the results of the interaction tests of seven SNPs (*p* = 0.004–0.045) are additional evidence for the role of *EPHX2* in AN, but show no correction for multiple testing. A small sample size (1205 cases and 1719 controls), a large case-control ratio (~3:1) in discovery, and mismatched ancestries between cases and controls (indicated by a principal component plot) may have also confounded the results. Although the authors acknowledge the need for additional replication, declaring the association between *EPHX2* and AN “statistically compelling” seems premature.

Another study examined whole-exome sequencing (WES) from four samples with multiple sclerosis (MS) selected from a family with more than 15 affected individuals [52]. The group found one novel missense mutation in the tyrosine kinase 2 (*TYK2*) gene, an MS-susceptibility locus established through GWAS [53,54]. The authors performed genotyping of the variant in all remaining family members and report the percentage of affected and unaffected individuals carrying the variant (10/14 (71.4%) and 28/60 (46.7%), respectively); the difference is not statistically significant (*p* = 0.17; not reported). Follow-up genotyping in an additional 2,104 cases and 1,543 controls revealed that the variant had a frequency of 0.8% in cases and 0.6% in controls, also not a statistically significant difference (which the authors state themselves). In fact, these frequencies are consistent with observed frequencies in several sets of healthy individuals [55]. Nonetheless, the authors conclude the variant has a modest effect on MS risk. Even though GWAS has established *TYK2* as an MS-associated locus, the particular variant implicated by this study is severely lacking in statistical evidence and seems highly unlikely to confer disease susceptibility.

Some sequencing studies rely on functional follow-up of a variant or gene in the absence of statistically compelling genetic evidence. One recent paper examining a family with 22 members with early-onset myocardial infarction (EOMI) implicated a frameshift insertion in the guanyl cyclase 1, soluble alpha 3 (*GUCY1A3*) gene and a nonsynonymous single nucleotide variant (SNV) in the chaperonin containing TCP1 (*CCT7*) gene in susceptibility to disease [56]. The frequency of the variants in affected *versus* unaffected family members was not statistically significant, as the sample size was small. A search for other susceptibility variants in these two genes in 252 EOMI cases and 800 controls yielded counts that were also not statistically significant (Fisher’s *p* = 0.023 and *p* = 0.12 for *GUCY1A3* and *CCT7*, respectively). Functional work on both variants is provided as additional evidence, indicating that mutations in both *GUCY1A3* and *CCT7* in mice induce a protein deficiency that can accelerate the formation of clots that potentially cause infarction. Guanyl cyclase is a key gene in signal transduction involved in vasodilation, and it is possible that these two loci influence susceptibility to EOMI. However, the statistical evidence from the genetic data alone is not strong enough to claim association between the mutations and the trait. Moreover, it is not immediately obvious whether the conclusions of functional work in mouse models will be pertinent to humans or whether findings from such an extreme family will be extendable to EOMI in the general population [57].

A WES study in sporadic amyotrophic lateral sclerosis (ALS) trios also relied on functional work to bolster the finding in the absence of statistically compelling results [58]. The group identified *de novo* mutations in 47 ALS patients and discovered 25 *de novo* variants in 25 different genes, a distribution consistent with the (null) distribution in healthy individuals [59]. A pathway analysis indicated enrichment for chromatin regulator genes, such as the synovial sarcoma translocation (*SS18L1* or *CREST*) gene, which contained a single *de novo* event in one sample. The authors found that variants in *CREST* inhibited neurite growth in animal models and claimed that *de novo* mutations in the gene confer ALS risk. However, a single *de novo* event from one individual, even if the variant is functional, is insufficient evidence to claim a role in disease susceptibility [60], and extensive replication efforts will be necessary to determine whether the ALS-*CREST* association is real. 

A similar WES effort in Hirschsprung’s disease sequenced two affected (related) samples and also used pathway analysis to investigate the role of the neuregulin 3 (*NRG3*) gene in disease susceptibility [61]. Three variants found in *NRG3* in the initial samples were followed up in 96 cases and 110 controls; the variants were carried by a few cases and no controls (Fisher’s *p* = 0.021, *p* = 0.22, *p* = 0.021, for the three variants, respectively). The authors discuss the biological plausibility of the *NRG3* association based on the gene’s role in the nervous system, but due to the small sample size, the genetic evidence for the association is lacking.

In each of these studies, the statistical evidence based on the provided data was insufficient. Although it is certainly possible that replication will show these findings to be real, their publication before gathering more persuasive statistical evidence from genetic data seems a symptom of a much larger problem. Researchers eager to publish novel results, peer reviewers not questioning the dearth of evidence, and journals enthusiastic to publish exciting stories that will garner both attention and citations all combine to allow findings that have not yet been demonstrated as statistically robust to enter scientific literature as such.

## 5. Conclusions: Moving Towards Permanent Change

A number of changes, addressing both technical challenges and cultural characteristics of research, can be implemented in order to improve the veracity of claims in published research. Though association testing approaches may differ between sequencing studies (such as selecting single-variant testing or gene-based testing), the universal application of particular thresholds will help to ensure the robustness of claimed associations. The statistical stringency of genome-wide significance at 5 × 10^−8^ has served the genetics community well and has ensured the robustness of the majority of GWAS findings. Single-variant testing across whole-genome data should maintain this threshold, if not establish a more conservative one given the increased number of variants tested. Similarly, burden tests of genes should be held to an exome-wide significance level, estimated to be approximately 5 × 10^−7^ [44]. Relaxing these thresholds, particularly tempting in the case of loss-of-function mutations or studies that analyze only a small set of genes, is unwise. A number of mechanisms make interpreting the true deleteriousness of a variant difficult; functional annotation alone does not guarantee a true loss-of-function mutation [44,48]. Findings from other association testing approaches, such as pathway analyses or polygenic modeling, should be evaluated based on appropriate Bonferroni correction for independent tests. Some may argue that it is overly conservative to hold a small set of genes or variants to an exome- or genome-wide threshold, but these smaller analyses are simply subsets of what will likely become exhaustive genome-wide searches. Contingent on the assembly of large datasets, testing every gene or variant is inevitable. False-positive signals can occur anywhere in the search space, and assuming these false positives will not occur in smaller, earlier searches is faulty logic [62].

**Figure 4:**
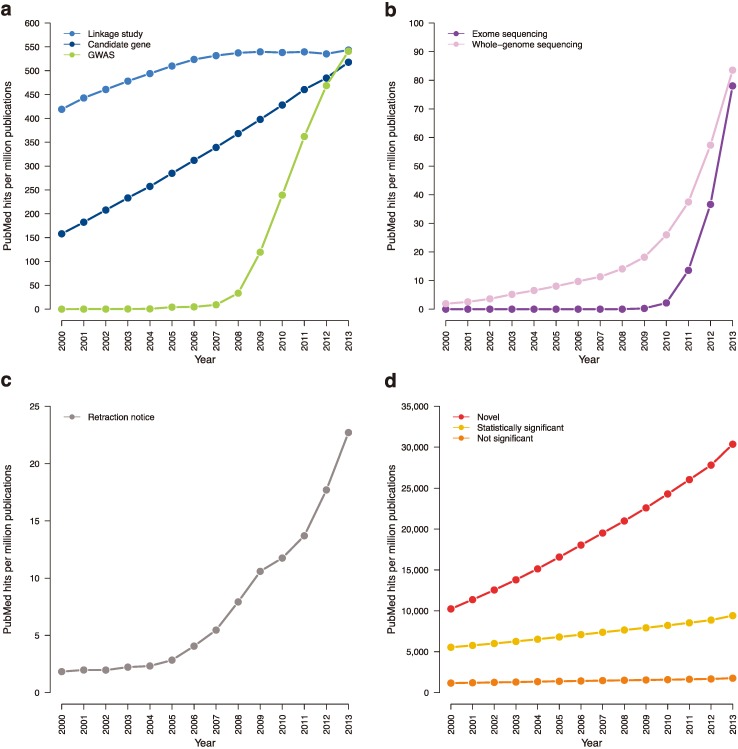
Trending topics on PubMed, 2000–2013. The cumulative number of times certain phrases appear per one million abstracts in PubMed. (**a**) Earlier genetic association studies (search terms: “candidate gene”; “linkage analysis” or “linkage study”; “genome-wide association study”). (**b**) Next-generation sequencing studies (search terms: “exome sequencing”; “whole-genome sequencing”). (**c**) Retraction notices (search terms: “retraction notice” or “notice of retraction”). (**d**) Claims of novelty and statistical significance (search terms: “novel”; “not statistically significant” or “not significant”; “statistically significant” removing “not statistically significant”).

Designing and performing a study correctly is also crucial to avoiding spurious associations. Large samples are of the utmost priority and will lead to additional discoveries, as demonstrated by a recent WES project in Alzheimer’s that discovered rare variants in the phospholipase D family (Member 3) (*PLD3*) gene by whole-exome sequencing of 14 families and replicated the finding in a large-scale cohort (11,000 European-ancestry cases and controls, gene burden odds ratio = 2.75, *p* = 1.44 × 10^−11^; 302 African-ancestry cases and controls, gene burden odds ratio = 5.48, *p* = 1.4 × 10^−3^) [63]. Leveraging population isolates [64] and assembling non-European samples [46] may also help in improving power, as was true in the GWAS era [65]. Because of varying study designs and technologies, standardizing NGS data processing and analysis is difficult and will likely take time as methods continue to develop. However, some best practices already exist [42,43,55] and should be followed. Further, the methods sections of papers should be as clear and explicit as possible, reading as “how-to” guides to allow peer reviewers to catch technical mistakes and aid external replication efforts. Ideally, data should be made publicly available whenever possible. Each of these steps will improve the quality of NGS studies as they are increasingly used in the future to study complex traits (Figure 4a,b).

Designing a sequencing study not only involves consideration of the technical aspects of the study but also of the analysis team. Diversifying the team to include scientists with an array of expertise will improve the study’s execution and increase the likelihood that the findings prove replicable. In addition to geneticists, computer scientists, statisticians, biologists, engineers, and clinicians can all contribute to different aspects of a sequencing study, from assembling the raw sequencing data to interpreting results. Methods for sequencing analysis are evolving rapidly, and multifaceted teams representing many scientific fields are optimal for keeping pace with this changing landscape.

Future scientific publications will also be improved by an open peer review system. Some journals, such as the journal of the European Molecular Biology Organization (EMBO), have already begun making peer review a public process to great success [66]. The exchange that occurs between scientists during peer review can be valuable not only for the authors, but also for other researchers. If reviews, responses and revisions are made public the scientific community will benefit as a whole by being able to design better studies and avoid errors that other researchers have made. Further, peer review is crucial to the scientific process, yet is rarely ever taught. Making peer review reports accessible to all can serve as a teaching tool, particularly for younger scientists who may be unfamiliar with the process [66].

Peer review is not the only aspect of publishing in scientific journals that should be open. Though some open-access journals were discredited in *Science*’s recent “sting”, several open-access journals have become highly reputable in the scientific community, and other journals are giving authors the option to make their article open-access. This trend towards open-access science should continue. Rather than hiding important discoveries behind exorbitantly priced subscriptions, journals should be making these findings easily accessible so that they may be discussed and retested by the scientific community and available to the public.

While changes such as statistical stringency, public peer review, and open-access articles are readily implementable, other changes in research culture will enhance the robustness of published findings, but will require a larger community effort. Journals should require researchers to increase transparency and meet certain criteria before submitting a manuscript for consideration. The Nature Publishing Group recently updated its editorial policies [67], requiring authors to fill out a checklist that accompanies their submitted manuscript. The checklist addresses areas of manuscripts, such as study design and analysis, which the editors have noticed are often not reported completely. The checklist also lends particular emphasis to justifying statistical analyses; the Nature Publishing Group will now consult with statisticians if there seem to be glaring analytic issues or if a referee suggests outside consultation. Other journals should adopt a similar strategy.

Retractions of genome-wide association and next-generation sequencing studies have been limited and should remain as such. To further increase the responsibility of journals in helping prevent retractions, the genetics community could establish a retraction index (similar to impact factor) for each journal. Such an index would track retraction rate and thus encourage journal editors to rigorously review findings and pursue a thorough peer-review process. Of course, researchers must take on greater responsibility for retractions, as well. Currently, retraction notices can be limited in information or completely ambiguous [34]. Instead, authors should be required to provide retraction notices that detail the steps that led to the retraction. The notice should be published as a brief article for the journal’s readership to see and appended to the original manuscript. Requiring a detailed notice will encourage authors to be particularly critical of their own work and may help deter future retractions (Figure 4c).

Journals should also work to rectify publication bias towards novel findings (Figure 4d). A study on *de novo* variation in Autism Spectrum Disorder (which did not discover any disease-susceptibility loci) extensively investigated population stratification in rare variants and the effects of different meta-analysis approaches on power [68]. A recent WES paper on Type 2 Diabetes also did not discover disease-associated genes, but performed a host of analyses to determine likely etiological architecture [69]. Such findings should be made accessible to the entire community, as they can clarify our understanding of the genetic architecture of complex traits and improve future studies. 

Journals can also be used to educate the scientific community in areas where it is lacking, such as applications of statistics and data interpretation. Three papers published together in the *Journal of the American Medical Association* explain how to interpret genome-wide association studies [70,71,72]. This fall, *Nature Methods* began publishing the column *Points of Significance* [73,74,75,76], which addresses important statistical concepts such as *p*-values, significance, and the relationship between sample size and power. *Nature* also recently ran a column that discussed the role of bias, the inexact nature of measurements, and the important distinction between correlation and causation [77]. Such articles do a great community service and will remain invaluable teaching tools to the many genetics researchers without formal training in statistics, allowing them to evaluate claims of novelty, both in their work and the work of others.

Of course, in addition to all of these larger changes, the field will hugely benefit from changes on a smaller scale. Senior scientists should encourage younger members of the field to conduct rigorous experiments, remain vigilant to prevent error, and seek additional help when in doubt about an analysis or result. Mistakes or “negative” findings should be met with discussion about how to improve a scientific question or refine an experiment. Randy Schekman, who won the Nobel Prize for physiology or medicine in 2013, has recently sparked public debate by announcing that he and all members of his lab will no longer be publishing in *Nature*, *Science*, and *Cell* [78]. Schekman’s announcement, while controversial because of its potentially detrimental effects on the careers of his younger lab members, has an important motivation: to publicly address the adverse effects that publication bias and lack of open-access have had on the field. Making younger scientists aware of the aspects of scientific culture that are damaging to the quality of published and public science and prompting them to push for change can only alter the field for the better.

Fortunately, science in general and genetics in particular has proven to be a highly adaptive field. Peer-review is a relatively new process, becoming standard practice for most scientific journals in the second half of the 20th century [79]. In less than a decade, the foundation of the Public Library of Science (PLoS) has helped to revolutionize publishing in science, providing the scientific community with a family of open-access journals and encouraging other journals to follow suit in its pursuit of freely available scientific literature [80]. In genetics specifically, the community acknowledged that candidate gene studies were poorly performing and established standards for more robust genome-wide association studies. Again, the tide has begun to shift as communities to discuss pre-prints continue to grow [81,82], researchers increasingly use social media to discuss many of the issues addressed here, and journals begin altering their editorial and publishing processes. With a concerted effort to improve published work, the field of human genetics is capable of permanent change.

## References

[1] Lander E.S., Linton L.M., Birren B., Nusbaum C., Zody M.C., Baldwin J., Devon K., Dewar K., Doyle M., FitzHugh W. (2001). Initial sequencing and analysis of the human genome. Nature.

[2] Clinton W.J. (2014). Remarks made by the President on the Completion of the First Survey of the Entire Human Genome Project.

[3] Hirschhorn J.N., Lohmueller K., Byrne E., Hirschhorn K. (2002). A comprehensive review of genetic association studies. Genet. Med..

[4] Hirschhorn J.N., Altshuler D. (2002). Once and again-issues surrounding replication in genetic association studies. J. Clin. Endocrinol. Metab..

[5] Ioannidis J.P., Ntzani E.E., Trikalinos T.A., Contopoulos-Ioannidis D.G. (2001). Replication validity of genetic association studies. Nat. Genet..

[6] Kathiresan S., Newton-Cheh C., Gerszten R.E. (2004). On the interpretation of genetic association studies. Eur. Heart J..

[7] Lohmueller K.E., Pearce C.L., Pike M., Lander E.S., Hirschhorn J.N. (2003). Meta-analysis of genetic association studies supports a contribution of common variants to susceptibility to common disease. Nat. Genet..

[8] Hirschhorn J.N., Daly M.J. (2005). Genome-wide association studies for common diseases and complex traits. Nat. Rev. Genet..

[9] The International HapMap Consortium (2005). A haplotype map of the human genome. Nature.

[10] Wang W.Y.S., Barratt B.J., Clayton D.G., Todd J.A. (2005). Genome-wide association studies: Theoretical and practical concerns. Nat. Rev. Genet..

[11] Dudbridge F., Gusnanto A. (2008). Estimation of significance thresholds for genomewide association scans. Genet. Epidemiol..

[12] Pe’er I., Yelensky R., Altshuler D., Daly M.J. (2008). Estimation of the multiple testing burden for genomewide association studies of nearly all common variants. Genet. Epidemiol..

[13] Price A.L., Patterson N.J., Plenge R.M., Weinblatt M.E., Shadick N.A., Reich D. (2006). Principal components analysis corrects for stratification in genome-wide association studies. Nat. Genet..

[14] Ioannidis J.P.A., Boffetta P., Little J., O’Brien T.R., Uitterlinden A.G., Vineis P., Balding D.J., Chokkalingam A., Dolan S.M., Flanders W.D. (2008). Assessment of cumulative evidence on genetic associations: interim guidelines. Int. J. Epidemiol..

[15] Kraft P., Zeggini E., Ioannidis J.P.A. (2009). Replication in genome-wide association studies. Stat. Sci..

[16] Chanock S.J., Manolio T., Boehnke M., Boerwinkle E., Hunter D.J., Thomas G., Hirschhorn J.N., Abecasis G., Altshuler D., Bailey-Wilson J.E. (2007). Replicating genotype-phenotype associations. Nature.

[17] Hindorff L.A., MacArthur J., Morales J., Junkins H.A., Hall P.N., Klemm A.K., Manolio T.A. A Catalog of Published Genome-Wide Association Studies. www.genome.gov/gwastudies/.

[18] Visscher P.M., Brown M.A., McCarthy M.I., Yang J. (2012). Five years of GWAS discovery. Am. J. Hum. Genet..

[19] Sebastiani P., Solovieff N., Puca A., Hartley S.W., Melista E., Andersen S., Dworkis D.A., Wilk J.B., Myers R.H., Steinberg M.H. (2010). Genetic signatures of exceptional longevity in humans. Science.

[20] Carmichael M. The little flaw in the longevity-gene study that could be a big problem. http://www.newsweek.com/little-flaw-longevity-gene-study-could-be-big-problem-74703/.

[21] Sebastiani P., Solovieff N., Puca A., Hartley S.W., Melista E., Andersen S., Dworkis D.A., Wilk J.B., Myers R.H., Steinberg M.H. (2011). Letters: Retraction. Science.

[22] (2013). Unreliable research: Trouble at the Lab. Economist.

[23] (2013). Problems with scientific reasearch: How science goes wrong. Economist.

[24] Neaves W. (2012). The roots of research misconduct. Nature.

[25] Macilwain C. (2012). The time is right to confront misconduct. Nature.

[26] Corbyn Z. (2012). Misconduct is the main cause of life-sciences retractions. Nature.

[27] Macilwain C. (2012). Scientific misconduct: More cops, more robbers?. Cell.

[28] Yong E., Ledford H., van Noorden R. (2013). Research ethics: 3 ways to blow the whistle. Nature.

[29] The Wellcome Trust Case Control Consortium (2007). Genome-wide association study of 14,000 cases of seven common diseases and 3000 shared controls. Nature.

[30] Zheng G., Freidlin B., Gastwirth J.L. (2006). Robust genomic control for association studies. Am. J. Hum. Genet..

[31] Clayton D.G., Walker N.M., Smyth D.J., Pask R., Cooper J.D., Maier L.M., Smink L.J., Lam A.C., Ovington N.R., Stevens H.E. (2005). Population structure, differential bias and genomic control in a large-scale, case-control association study. Nat. Genet..

[32] Plagnol V., Cooper J.D., Todd J.A., Clayton D.G. (2007). A method to address differential bias in genotyping in large-scale association studies. PLoS Genet..

[33] Steen R.G. (2011). Retractions in the scientific literature: Is the incidence of research fraud increasing?. J. Med. Ethics.

[34] Fang F.C., Steen R.G., Casadevall A. (2012). Misconduct accounts for the majority of retracted scientific publications. Proc. Natl. Acad. Sci. USA.

[35] Ioannidis J.P.A. (2005). Why most published research findings are false. PLoS Med..

[36] Young N.S., Ioannidis J.P.A., Al-Ubaydli O. (2008). Why current publication practices may distort science. PLoS Med..

[37] Ioannidis J.P.A. (2006). Concentration of the most-cited papers in the scientific literature: Analysis of journal ecosystems. PLoS One.

[38] Bjornshauge L., Brage S., Mitchell D., Zeylon R. Directory of Open Access Journals. www.doaj.org/.

[39] Bohannon J. (2013). Who’s afraid of peer review?. Science.

[40] Stahl E.A., Wegmann D., Trynka G., Gutierrez-Achury J., Do R., Voight B.F., Kraft P., Chen R., Kallberg H.J., Kurreeman F.A.S. (2012). Bayesian inference analyses of the polygenic architecture of rheumatoid arthritis. Nat. Genet..

[41] Hemani G., Yang J., Vinkhuyzen A., Powell J.E., Willemsen G., Hottenga J.-J., Abdellaoui A., Mangino M., Valdes A.M., Medland S.E. (2013). Inference of the genetic architecture underlying BMI and height with the use of 20,240 sibling pairs. Am. J. Hum. Genet..

[42] DePristo M.A., Banks E., Poplin R., Garimella K.V., Maguire J.R., Hartl C., Philippakis A.A., del Angel G., Rivas M.A., Hanna M. (2011). A framework for variation discovery and genotyping using next-generation DNA sequencing data. Nat. Genet..

[43] McKenna A., Hanna M., Banks E., Sivachenko A., Cibulskis K., Kernytsky A., Garimella K., Altshuler D., Gabriel S., Daly M. (2010). The Genome Analysis Toolkit: A MapReduce framework for analyzing next-generation DNA sequencing data. Genome Res..

[44] Do R., Kathiresan S., Abecasis G.R. (2012). Exome sequencing and complex disease: Practical aspects of rare variant association studies. Hum. Mol. Genet..

[45] Mathieson I., McVean G. (2012). Differential confounding of rare and common variants in spatially structured populations. Nat. Genet..

[46] Kiezun A., Garimella K., Do R., Stitziel N.O., Neale B.M., McLaren P.J., Gupta N., Sklar P., Sullivan P.F., Moran J.L. (2012). Exome sequencing and the genetic basis of complex traits. Nat. Genet..

[47] Li B., Leal S.M. (2009). Discovery of rare variants via sequencing: Implications for the design of complex trait association studies. PLoS Genet..

[48] MacArthur D.G., Balasubramanian S., Frankish A., Huang N., Morris J., Walter K., Jostins L., Habegger L., Pickrell J.K., Montgomery S.B. (2012). A systematic survey of loss-of-function variants in human protein-coding genes. Science.

[49] Kryukov G.V., Shpunt A., Stamatoyannopoulos J.A., Sunyaev S.R. (2009). Power of deep, all-exon resequencing for discovery of human trait genes. Proc. Natl. Acad. Sci. USA.

[50] Chapman J.M., Cooper J.D., Todd J.A., Clayton D.G. (2003). Detecting disease associations due to linkage disequilibrium using haplotype tags: A class of tests and the determinants of statistical power. Hum. Hered..

[51] Scott-Van Zeeland A.A., Bloss C.S., Tewhey R., Bansal V., Torkamani A., Libiger O., Duvvuri V., Wineinger N., Galvez L., Darst B.F. (2013). Evidence for the role of EPHX2 gene variants in anorexia nervosa. Mol. Psychiatry.

[52] Dyment D.A., Cader M.Z., Chao M.J., Lincoln M.R., Morrison K.M., Disanto G., Morahan J.M., de Luca G.C., Sadovnick A.D., Lepage P. (2012). Exome sequencing identifies a novel multiple sclerosis susceptibility variant in the TYK2 gene. Neurology.

[53] Ban M., Goris A., Lorentzen A.R., Baker A., Mihalova T., Ingram G., Booth D.R., Heard R.N., Stewart G.J., Bogaert E. (2009). Replication analysis identifies TYK2 as a multiple sclerosis susceptibility factor. Eur. J. Hum. Genet..

[54] Australia and New Zealand Multiple Sclerosis Genetics Consortium (2009). Genome-wide association study identifies new multiple sclerosis susceptibility loci on chromosomes 12 and 20. Nat. Genet..

[55] Abecasis G.R., Auton A., Brooks L.D., DePristo M.A., Durbin R.M., Handsaker R.E., Kang H.M., Marth G.T., McVean G.A. (2012). An integrated map of genetic variation from 1092 human genomes. Nature.

[56] Erdmann J., Stark K., Esslinger U.B., Rumpf P.M., Koesling D., de Wit C., Kaiser F.J., Braunholz D., Medack A., Fischer M. (2013). Dysfunctional nitric oxide signalling increases risk of myocardial infarction. Nature.

[57] Flannick J., Beer N.L., Bick A.G., Agarwala V., Molnes J., Gupta N., Burtt N.P., Florez J.C., Meigs J.B., Taylor H. (2013). Assessing the phenotypic effects in the general population of rare variants in genes for a dominant Mendelian form of diabetes. Nat. Genet..

[58] Chesi A., Staahl B.T., Jovičić A., Couthouis J., Fasolino M., Raphael A.R., Yamazaki T., Elias L., Polak M., Kelly C. (2013). Exome sequencing to identify *de novo* mutations in sporadic ALS trios. Nat. Neurosci..

[59] Neale B.M., Kou Y., Liu L., Ma’ayan A., Samocha K.E., Sabo A., Lin C.-F., Stevens C., Wang L.-S., Makarov V. (2012). Patterns and rates of exonic *de novo* mutations in autism spectrum disorders. Nature.

[60] Gratten J., Visscher P.M., Mowry B.J., Wray N.R. (2013). Interpreting the role of de novo protein-coding mutations in neuropsychiatric disease. Nat. Genet..

[61] Yang J., Duan S., Zhong R., Yin J., Pu J., Ke J., Lu X., Zou L., Zhang H., Zhu Z. (2013). Exome sequencing identified NRG3 as a novel susceptible gene of Hirschsprung’s disease in a Chinese population. Mol. Neurobiol..

[62] Lander E., Kruglyak L. (1995). Genetic dissection of complex traits: Guidelines for interpreting and reporting linkage results. Nat. Genet..

[63] Cruchaga C., Karch C.M., Jin S.C., Benitez B.A., Cai Y., Guerreiro R., Harari O., Norton J., Budde J., Bertelsen S. (2013). Rare coding variants in the phospholipase D3 gene confer risk for Alzheimer’s disease. Nature.

[64] Panoutsopoulou K., Tachmazidou I., Zeggini E. (2013). In search of low-frequency and rare variants affecting complex traits. Hum. Mol. Genet..

[65] Pulit S.L., Voight B.F., de Bakker P.I.W. (2010). Multiethnic genetic association studies improve power for locus discovery. PLoS One.

[66] Pulverer B. (2010). Transparency showcases strength of peer review. Nature.

[67] Nature Neuroscience Editors (2013). Raising standards. Nat. Neurosci..

[68] Liu L., Sabo A., Neale B.M., Nagaswamy U., Stevens C., Lim E., Bodea C.A., Muzny D., Reid J.G., Banks E. (2013). Analysis of rare, exonic variation amongst subjects with autism spectrum disorders and population controls. PLoS Genet..

[69] Lohmueller K.E., Sparsø T., Li Q., Andersson E., Korneliussen T., Albrechtsen A., Banasik K., Grarup N., Hallgrimsdottir I., Kiil K. (2013). Whole-exome sequencing of 2000 danish individuals and the role of rare coding variants in type 2 diabetes. Am. J. Hum. Genet..

[70] Attia J., Ioannidis J.P.A., Thakkinstian A., McEvoy M., Scott R.J., Minelli C., Thompson J., Infante-Rivard C., Guyatt G. (2009). How to use an article about genetic association: A: Background concepts. JAMA.

[71] Attia J., Ioannidis J.P.A., Thakkinstian A., McEvoy M., Scott R.J., Minelli C., Thompson J., Infante-Rivard C., Guyatt G. (2009). How to use an article about genetic association: B: Are the results of the study valid?. JAMA.

[72] Attia J., Ioannidis J.P.A., Thakkinstian A., McEvoy M., Scott R.J., Minelli C., Thompson J., Infante-Rivard C., Guyatt G. (2009). How to use an article about genetic association: C: What are the results and will they help me in caring for my patients?. JAMA.

[73] Krzywinski M., Altman N. (2013). Points of significance: Importance of being uncertain. Nat. Methods.

[74] Krzywinski M., Altman N. (2013). Points of significance: error bars. Nat. Methods.

[75] Krzywinski M., Altman N. (2013). Points of significance: Significance, P values and t-tests. Nat. Methods.

[76] Krzywinski M., Altman N. (2013). Points of significance: Power and sample size. Nat. Methods.

[77] Sutherland W.J., Spiegelhalter D., Burgman M.A. (2013). Policy: Twenty tips for interpreting scientific claims. Nature.

[78] Schekman R. “How journals like Nature, Cell and Science are damaging science.”. http://www.theguardian.com/commentisfree/2013/dec/09/how-journals-nature-science-cell-damage-science/.

[79] Chapelle F.H. (2014). The history and practice of peer review. Ground Water.

[80] Van Noorden R. (2013). Open access: The true cost of science publishing. Nature.

[81] Coop G., Howie B., Pickrell J. Haldane’s Sieve. haldanessieve.org/.

[82] Cornell University Library. arXiv. www.arxiv.org/.

